# Congenital Melanotic Macule of the Tongue: A Five-Year Follow-Up

**DOI:** 10.5826/dpc.1104a122

**Published:** 2021-10-01

**Authors:** Natália Maira Resende, Flávia Vasques Bittencourt, Bernardo Gontijo

**Affiliations:** 1 Federal University of Minas Gerais Medical School, Belo Horizonte, MG, Brazil

**Keywords:** nevus, pigmented, melanoma, dermoscopy

## Introduction

Congenital melanotic macule of the tongue (CMMT) is a rare and underestimated benign entity with few cases reported in the literature [1]. Clinical characteristics include solitary or multiple asymptomatic congenital melanotic lesions on the tongue with subsequent proportional growth; diameter ranging from 0.3 to 3 cm; homogeneous or heterogeneous color, often very dark and worrisome; and a negative family history of systemic conditions associated with mucosal pigmentation. CMMT can occur in all skin phototypes, but it tends to be more frequent in dark-skinned patients. Histologic findings show increased deposits of melanin in the basal cell layer, with a normal number of melanocytes and various degrees of hyperkeratosis.

Other diseases presenting oral mucous hyperpigmentation, such as pigmented fungiform papillae, lingua nigra villosa, Laugier Hunziker syndrome, and Peutz–Jeghers syndrome, can easily be excluded based on clinical grounds alone. Oral congenital melanocytic nevi are exceptionally rare, with only 6 cases reported in the literature, in all cases the tongue was not involved [2]. Although CMMT clinical features can be worrisome and mimic melanoma, this malignancy is exceptionally rare in childhood and has never been reported in the oral cavity in this age group [1].

The cause of CMMT is unclear. Congenital lesions might represent a hamartoma of melanocytes with localized functional change in melanin production. No predisposing or causative factors occurring during gestation have been linked to CMMT.

To exclude malignancy, some authors suggest performing a tongue biopsy. Others argue that follow-up alone is justified, since CMMT clinical features are distinctive, congenital melanocytic nevus of the tongue is an extremely rare disorder, and congenital melanoma has never been reported in the oral cavity. If significant changes occur, a biopsy should be considered.

## Case Presentation

**Figure 1:**
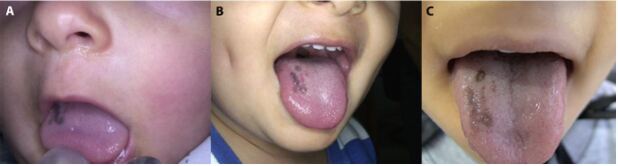
Clinical presentations. (A) Five months old. (B) Two years old. (C) Five years old.

**Figure 2:**
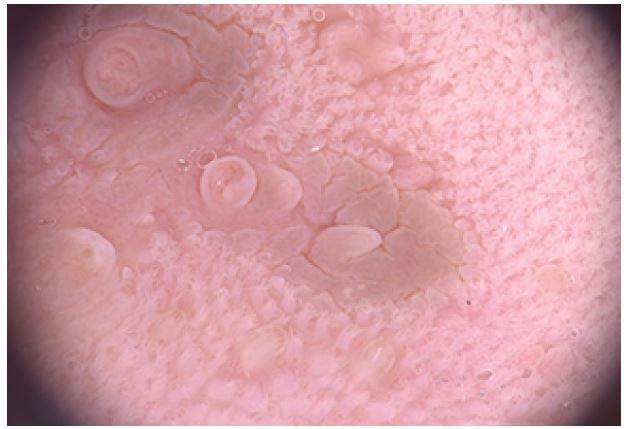
Dermoscopy

A 5-month-old boy, born at term, skin phototype III, was referred for evaluation of congenital, asymptomatic pigmented lesions on the tongue that had enlarged proportionally to the infant’s growth. Figures 1 A–C show clinical presentations upon the patient’s first visit, and at the age of 2 and 5 years, respectively. His medical history was unremarkable and there was no family history of melanoma or pigmented oral lesions. Physical examination revealed asymmetrical brown-gray macules located on the right side of the dorsal surface of the tongue. No palpable cervical nodes were found. Due to the child’s compliance, dermoscopy was only carried out at 2 years of age. Oral dermoscopy descriptions are rare, and most focus on labial lesions. As for the tongue, the few existing reports describe the patterns of pigmented fungiform papillae. We identified a homogeneous light brown area and projections with vessels (Figure 2). Mucous melanoma shows a great heterogeneity of colors and a multicomponent pattern, both of which were absent in our patient.

## Conclusions

Our patient’s follow-up, with photographic documentation from the first medical evaluation at 5 months until 5 years of age, is one of the longest reported in the literature. Additionally, this is the first dermoscopy report in a case of CMMT. Although this entity is more common in people with dark phototypes (IV, V e VI), our case reports CMMT in a phototype III child.

It is important to raise awareness on this underreported entity to avoid unnecessary aggressive surgical approaches in children. Clinical follow-up alone is warranted due to the extremely rare occurrence of oral congenital melanocytic nevi and the unreported childhood congenital melanoma of the oral cavity.
